# The Risk of Non-arteritic Ischemic Optic Neuropathy Post-intravitreal Bevacizumab Injection

**DOI:** 10.7759/cureus.30185

**Published:** 2022-10-11

**Authors:** Nasser A Fugara, Zaineh A Shawareb, Nancy K Rakkad, Manar L Barhoum, Bana A Shawareb, Myrna M Al-Madani, Mousa V Al-Madani

**Affiliations:** 1 Ophthalmology, King Hussein Medical Center, Royal Medical Services of Jordanian Armed Forces, Amman, JOR

**Keywords:** non-arteritic ischemic optic neuropathy, hypertension, bevacizumab, intravitreal, diabetes mellitus

## Abstract

Objectives: To report the incidence of non-arteritic ischemic optic neuropathy in diabetic patients treated with intravitreal bevacizumab injection.

Methods: A prospective comparative analytic study was done in the King Hussein Medical Center during the period between June 2020 and June 2021. Inclusion criteria included diabetic patients who attended a retina clinic. Exclusion criteria included patients with pre-existing ischemic optic neuropathy. Patients were divided into two groups. The first group included diabetic patients who did not require bevacizumab injection and were treated either with follow-up visits or pan-retinal photocoagulation, and the second group included patients who were treated with intravitreal bevacizumab. Patients were followed up for a period of one year. Data collected in two groups included the total number of patients and the number of patients with non-arteritic ischemic optic neuropathy. Results were compared in both groups. A P-value was used to study the statistical significance and was considered to be statistically significant if ≤0.05.

Results: The mean age for patients in group 1 was 64.3 years, with a male to female ratio of 1.1 to 1. In group 2, the mean age was 66.2 years, with a male to female ratio of 1.2 to 1. The total number of patients in group 1 was 7375, among whom 68 patients had non-arteritic ischemic optic neuropathy. While in group 2, the total number was 2468 and 49 of them had non-arteritic ischemic optic neuropathy. Most cases of non-arteritic ischemic optic neuropathy were seen in patients who had received three or more injections.

Conclusion: Intravitreal bevacizumab in diabetic patients is considered a risk factor for the development of non-arteritic ischemic optic neuropathy, especially in patients receiving more than three injections.

## Introduction

Non-arteritic ischemic optic neuropathy (NAION) occurs due to decreased blood supply to the optic nerve head. It is the most common cause of optic disc swelling in adults over 50 years of age [1,2]. The exact mechanism causing reduced blood flow to the optic disc is not proven, but there are many risk factors that are strongly associated with NAION, such as hypertension, diabetes mellitus, hyperlipidemia, coronary artery disease, left ventricular hypertrophy, atrial fibrillation, and chronic obstructive pulmonary disease [3-5]. In addition, patients undergoing cataract surgery are at a higher risk of developing NAION [4].

Bevacizumab is a recombinant humanized monoclonal IgG1 antibody that binds to and inhibits vascular endothelial growth factor (VEGF), reducing the growth of new blood vessels [6]. It is extensively used off-label as an intravitreal injection for the treatment of many eye diseases, such as diabetic macular edema and wet age-related macular degeneration [7]. Although its complications are very rare, some of them are elevated intraocular pressure, retinal pigment epithelium tear, endophthalmitis, and retinal detachment [8,9].

In this study, we aimed to investigate the association between intravitreal bevacizumab and non-arteritic ischemic optic neuropathy at the King Hussein Medical Center of the Royal Medical Services.

## Materials and methods

A prospective comparative analytic study was done in the King Hussein Medical Center during the period between June 2020 and June 2021. Inclusion criteria included diabetic patients who attended the retina clinic. An ophthalmologic examination included Snellen’s best corrected visual acuity, anterior segment examination, applanation tonometry, optic nerve function assessment, and retinal and optic disc examination after dilated fundoscopy with a +78 lens. Exclusion criteria included patients with pre-existing ischemic optic neuropathy. The retina clinic at the King Hussein Medical Center is a tertiary center, and all diabetic patients referred there have diabetic retinopathy.

Patients were categorized according to the stage of retinopathy, either proliferative or non-proliferative and according to the presence of clinically significant macular edema. The decision on whether to observe or treat by intravitreal injection or laser was made by a retina specialist according to the protocol used in our hospital. Patients with mild to moderate diabetic retinopathy without diabetic macular edema were observed. Patients with diabetic macular edema involving the center were treated by intravitreal injection, and patients with edema sparing the center were treated by focal laser. In the case of focal laser failure, the intravitreal injection was used. Pan-retinal photocoagulation was used in proliferative diabetic retinopathy. Patients were divided into two groups; a control group (group 1) included diabetic patients who did not require bevacizumab injection and were treated either with follow-up visits or pan-retinal photocoagulation and a study group (group 2) included patients who were treated with intravitreal bevacizumab.

Our study is not a randomized control study; thus, the two groups were not equal in number as patients were categorized into corresponding groups according to the modality of treatment they required. We compared disease severity among the two groups depending on HbA1C level and other co-morbidities control, such as blood pressure and hyperlipidemia, and the presence of obstructive sleep apnea. Patients were followed up for a period of one year. The first visit was one week after each injection and then once every six weeks unless further injections were required. Patients who were suspected to have NAION were referred to the neuro-ophthalmology clinic where an optic nerve assessment including pupillary examination, color vision, optic disc appearance, and 24-2 Humphrey's visual field was done. All patients with NAION field defects were detected by 24-2 Humphrey's visual field test. 30-2 Humphrey's visual field test was done when an optic nerve insult was still suspected and not proven by clinical examination and 24-2 Humphrey's visual field test. Data collected in two groups included the total number of patients and the number of patients with NAION. The results were compared in both groups taking into consideration the presence of other risk factors such as hypertension and hyperlipidemia and local risk factors that included previous cataract surgery, crowded disc, small cup-to-disc ratio, age-related macular degeneration, and retinal vascular disease. The number of injections in group 2 was recorded. Also, we recorded the onset of the occurrence of optic neuropathy in relation to the time of injection. A P-value was used to study the statistical significance and was considered to be statistically significant if ≤0.05. Consent form was obtained from the ethical committee of the Royal Medical Services, IRB number 11/72022.

## Results

A total of 9843 diabetic patients were enrolled in the study. Almost a quarter of them (2468 patients) were treated with intravitreal bevacizumab. Table 1 shows the age and gender distribution of patients in both groups. It also shows whether patients had controlled diabetes, hypertension, hyperlipidemia, or obstructive sleep apnea, which are considered risk factors for the development of NAION, and whether there are any local risk factors, such as retinal vascular disease, crowded optic disc, and previous cataract surgery.

**Table 1:** Age, gender distribution for both groups, and the presence of risk factors for ischemic optic neuropathy.

Risk factor		Group 1	Group 2
Age		64.3	66.2
Gender (male:female) ratio		1.1:1	1.2:1
HbA1C less than 5.7%		298 (40%)	103 (41.7%)
Presence of hypertension	Controlled	782 (31.7%)	2440 (33.1%)
	Uncontrolled	199 (8%)	610 (8.3%)
	Total	981 (39.7%)	3050 (41.4%)
Presence of hyperlipidemia	Controlled	106 (4.3%)	371 (5%)
	Uncontrolled	25 (1%)	65 (0.7%)
	Total	131 (5.3%)	436 (5.7%)
Patients diagnosed with obstructive sleep apnea		82 (1.1%)	25 (1%)
Local risk factors		870 (11.8%)	250 (10.1%)

The mean age for patients in group 1 was 64.3 years, with a male-to-female ratio of 1.1 to 1, and 66.2 years in group 2, with a male-to-female ratio of 1.2 to 1 (Table 1). Other medical risk factors for NAION did not show a statistically significant difference between the two groups. Although both groups were not equal in number, there was no statistically significant difference in disease severity among the two groups as we compared HbA1C level, blood pressure and hyperlipidemia control, and the presence of obstructive sleep apnea as risk factors for NAION development (Table 1).

Table 2 shows the total number of patients in both groups and the total number of patients who developed NAION. The total number of patients in group 1 was 7375, among them 68 patients (0.92%) had NAION. In group 2, the total number of patients was 2468, and among them 49 patients (1.99%) had NAION.

**Table 2:** Total number of patients and number of patients with ischemic optic neuropathy in both groups. NAION: non-arteritic ischemic optic neuropathy.

Category	Group 1	Group 2	P-value
Total number of patients	7375	2468	-
NAION	68	49	0.02 < p < 0.05
Percentage	0.92%	1.99%	-

The vast majority of patients with NAION in group 2 received three injections or more (31 patients), as shown in Table 3. Forty-one patients had the onset of NAION in the first week after receiving the injection, twenty-nine of them had their third injection, eleven of them had their second injection, and one had his first injection.

**Table 3:** Number of injections in group 2 and the onset of NAION post-injection. NAION: non-arteritic ischemic optic neuropathy.

Criteria		Number of patients with NAION	P-value
Number of injections	One	4	-
	Two	14	0.02 < p < 0.05
	Three or more	31	P < 0.05
Onset of NAION post-injection	Within one week	41	P < 0.01
	After one week till one month	7	P < 0.05
	More than one month	1	-

## Discussion

The exact mechanism of NAION is still not proven, but studies have proven many risk factors that are associated with it. Literature reports that diabetes mellitus, hypertension, and hyperlipidemia are major risk factors for developing non-arteritic ischemic optic neuropathy [3,5,10,11].

There is controversy in the literature about whether NAION can be attributed to anti-vascular endothelial factor (VEGF) injections [12-16]. On the contrary, anti-VEGF remains a proven method of treating patients with cystoid macular edema and diabetic retinopathy [7,8]. Paradoxically, some studies showed that anti-VEGF is beneficial in the treatment of NAION [14,17]. 

In our study, we compared two groups of diabetic patients known to have diabetic retinopathy who attended a retina clinic at King Hussein Medical Center. Patients were evaluated by a retina specialist to determine the treatment plan, whether they did not need treatment or needed treatment in the form of laser or intravitreal anti-VEGF injection. Patients with suspected optic nerve dysfunction were referred to a neuro-ophthalmology clinic where patients with confirmed NAION were excluded from the study. The first group was not treated with bevacizumab, while the second group received one injection or more. During the follow-up period, any patient with suspected NAION was referred to the neuro-ophthalmology clinic to confirm the diagnosis. Optic nerve function was done, including pupillary examination, color vision, optic disc appearance, and 24-2 Humphrey's visual field. We evaluated whether both groups showed statistically significant differences regarding age, gender, and risk factors and found that there was no statistically significant difference between the two groups (Table 1). The mean age for group 1 was 64.3 years and for group 2 was 66.2 years. The male to female ratio was 1.1 to 1 and 1.2 to 1 in the two groups, respectively. In addition, there was no statistically significant difference regarding disease and co-morbidity presence and severity between the two groups. The HbA1C level was less than 5.7% in 40% of patients in group 1 and 41.7% of patients in group 2. Hypertension was seen in 41.4% of patients in group 1, compared to 39.7% of patients in group 2. It was controlled by 33.1% and 31.7% of the two groups, respectively. Hyperlipidemia was seen in 5.7% of group 1 and 5.3% of group 2 and was controlled in 5% and 4.3% of the two groups. Obstructive sleep apnea was diagnosed in 82 patients (1.1%) of group 1 and 25 patients (1%) of group 2. Local risk factors were seen in 11.8% of patients in group 1 and 10.1% of patients in group 2. Reported local factors to cause NAION are previous cataract surgery [4], crowded disc [18], the small cup-to-disc ratio [19], age-related macular degeneration, and retinal vascular disease [11] did not show a statistically significant difference of 11.8% versus 10.1 % (Table 1). The commonest local risk factor we found was previous cataract surgery, which was seen in the majority of patients with local risk factors. It was seen in 742 patients in group 1 and 211 patients in group 2, followed by age-related macular degeneration, which was seen in 82 and 27 patients in the two groups, respectively.

A total of 9843 patients were enrolled in our study. Almost 75% of them (7375 patients) were treated by either observation or laser, of which NAION occurred in 68 patients (0.92%). As mentioned earlier, patients who required observation only had diabetic retinopathy in its early stage. It is reported that diabetes raises the risk of NAION by 40% in adults above 67 years [10]. In group 2, NAION occurred in 49 patients forming 1.99% of the 2468 patients in the group. Although the percentage is more than double that of the control group, the figure was not statistically significant as shown in Table 2 (0.02 < p < 0.05).

Analysis of the occurrence of NAION in relation to the number of injections was studied as well. Table 3 shows that the vast majority of patients with NAION (45 patients, 91.8%) received two or more injections, 31 patients (63.3%) received three or more injections, 14 patients (28.6%) received two injections, and only four patients (8.2%) received one injection. Evaluating the statistical significance, the risk of NAION was statistically significant for patients who received three or more injections (p < 0.05). In addition to multiple injections, such a category of patients usually has less controlled diabetes, which further increases the risk of developing NAION. Chen et al. reported that patients with age-related macular degeneration who received a higher number of intravitreal anti-VEGF injections were at a higher risk for developing NAION [20]. Table 3 also shows the onset of NAION in relation to the time of injection. Only one patient developed NAION after one month of the injection, which could be possibly related to the pre-existence of uncontrolled diabetes and hypertension in that patient and possibly not related to the injection itself. The vast majority of NAION occurred in the first-week post-injection (41 patients) and was statistically significant (p < 0.01). Seven patients developed NAION after the first week of injection and within the first month (Table 3).

The limitation of our study was the inability to attribute the occurrence of NAION to intravitreal bevacizumab injection or the presence of risk factors such as diabetes, hypertension, and hyperlipidemia. Another limitation is whether the occurrence of NAION, especially in patients who required multiple injections, could be attributed to the severity of the disease itself, poorly controlled diabetes and co-morbidities, or the number of injections. In order to minimize this limitation, we investigated the pre-existence of risk factors as well as disease and co-morbidity severity in both groups and evaluated if there was any statistically significant difference between them.

## Conclusions

Intravitreal anti-VEGF treatment with bevacizumab for diabetic retinopathy remains an effective and golden modality of treatment. On the other hand, intravitreal bevacizumab in diabetic patients is possibly a risk factor for the development of non-arteritic ischemic optic neuropathy. Based on the results we obtained, we consider three or more injections of intravitreal anti-VEGF as a major risk factor for developing NAION. In addition to multiple injections, this category of patients usually has less controlled diabetes, which further increases the risk of developing NAION. It is important to look for NAION, especially in the first-week post-injection. We advise monitoring those patients with multiple risk factors, either medical or local, for the development of NAION, especially those receiving three injections or more. 
